# Targeting MTA1/HIF‐1*α* signaling by pterostilbene in combination with histone deacetylase inhibitor attenuates prostate cancer progression

**DOI:** 10.1002/cam4.1209

**Published:** 2017-10-10

**Authors:** Nasir A. Butt, Avinash Kumar, Swati Dhar, Agnes M. Rimando, Israh Akhtar, John C. Hancock, Janice M. Lage, Charles R. Pound, Jack R. Lewin, Christian R. Gomez, Anait S. Levenson

**Affiliations:** ^1^ Cancer Institute University of Mississippi Medical Center Jackson Mississippi; ^2^ Department of Pathology University of Mississippi Medical Center Jackson Mississippi; ^3^ Arnold & Marie Schwartz College of Pharmacy and Health Sciences Long Island University Brooklyn New York; ^4^ Department of Radiation Oncology University of Mississippi Medical Center Jackson Mississippi; ^5^ United State Department of Agriculture Agriculture Research Service Natural Product Utilization Research Unit University Mississippi; ^6^ Division of Urology Department of Surgery University of Mississippi Medical Center Jackson Mississippi

**Keywords:** Combination strategy, MTA1, prostate cancer, *Pten* knockout mice, pterostilbene, SAHA

## Abstract

The metastasis‐associated protein 1(MTA1)/histone deacetylase (HDAC) unit is a cancer progression‐related epigenetic regulator, which is overexpressed in hormone‐refractory and metastatic prostate cancer (PCa). In our previous studies, we found a significantly increased MTA1 expression in a prostate‐specific *Pten*‐null mouse model. We also demonstrated that stilbenes, namely resveratrol and pterostilbene (Pter), affect MTA1/HDAC signaling, including deacetylation of tumor suppressors p53 and PTEN. In this study, we examined whether inhibition of MTA1/HDAC using combination of Pter and a clinically approved HDAC inhibitor, SAHA (suberoylanilide hydroxamic acid, vorinostat), which also downregulates MTA1, could block prostate tumor progression *in vivo*. We generated and utilized a luciferase reporter in a prostate‐specific *Pten*‐null mouse model (*Pb‐Cre* ^+^; *Pten* ^f/f^; *Rosa26* ^*Luc*/+^) to evaluate the anticancer efficacy of Pter/SAHA combinatorial approach. Our data showed that Pter sensitized tumor cells to SAHA treatment resulting in inhibiting tumor growth and additional decline of tumor progression. These effects were dependent on the reduction of MTA1‐associated proangiogenic factors HIF‐1*α*, VEGF, and IL‐1*β* leading to decreased angiogenesis. In addition, treatment of PCa cell lines *in vitro* with combined Pter and low dose SAHA resulted in more potent inhibition of MTA1/HIF‐1*α* than by high dose SAHA alone. Our study provides preclinical evidence that Pter/SAHA combination treatment inhibits MTA1/HIF‐1*α* tumor‐promoting signaling in PCa. The beneficial outcome of combinatorial strategy using a natural agent and an approved drug for higher efficacy and less toxicity supports further development of MTA1‐targeted therapies in PCa.

## Introduction

Prostate cancer (PCa) is the second most common cause of cancer‐related death in men in the USA because of advanced castrate‐resistant disease and associated metastasis. Since the incidence of this slow growing cancer is very high and diet is an associated risk factor 1, efforts have been driven toward the development of chemopreventive strategies with the use of natural bioactive compounds 2, 3, 4. Our group has shown that stilbenes, particularly resveratrol and pterostilbene found in grapes and blueberries, have targeted chemo‐protective effects against PCa *in vitro* and *in vivo* 5, 6, 7, 8, 9, 10, 11, 12, 13, 14, 15. We have shown that resveratrol exerted its anticancer activity through inhibiting metastasis‐associated protein 1 (MTA1) and destabilizing MTA1/HDAC (histone deacetylases 1 and 2) unit of the nucleosome remodeling and deacetylase (NuRD) multiprotein complex, which is involved in chromatin remodeling and gene silencing 2, 5, 6, 15. From a therapeutic perspective, dietary agents are being intensively studied for their chemosensitizing targeted anticancer properties to achieve an enhanced efficacy 16, 17, 18, 19. In our previous study, we have shown that combination of resveratrol and clinically approved HDAC inhibitor SAHA (suberoylanilide hydroxamic acid, vorinostat), synergistically increased p53 acetylation resulting in significantly increased apoptosis compared to each agent alone 15. While one of the well‐characterized biochemical effects of SAHA is inhibition of HDAC activity 20, 21, 22, we have also observed downregulation of MTA1 levels in PCa cells treated with HDAC inhibitors 12. Combinatorial strategies using SAHA in PCa warrants further investigation since it has been shown to be effective in inhibiting PCa cell growth in culture and xenografts 23 and in combination with androgen receptor antagonists 24. Vorinostat was tested in a phase I clinical trial in combination with doxorubicin 25 and it has been used in a phase II trial as a second‐line therapy for castrate‐resistant PCa 26. On the other hand, our finding of MTA1/HDAC as a new molecular target of resveratrol and its potent analogs, particularly pterostilbene (Pter) 2, 5, 6, 7, 10, 12, 15, opens opportunities for further preclinical validation of Pter not only as a chemopreventive agent but also as a candidate for novel combinatorial therapeutic approaches.

In our previous studies, we have found MTA1 overexpression as a novel factor that facilitates inflammation‐associated cancer initiation, epithelial‐to‐mesenchymal transition (EMT), and PCa progression associated with angiogenesis, invasiveness, and metastatic potential 10, 14. In addition to our previous finding of MTA1′s association with angiogenesis *in vitro* and in xenografts 14, we have recently demonstrated that pharmacological inhibition of highly elevated MTA1 expression and its angiogenesis‐associated network by Pter, resulted in chemopreventive and therapeutic efficacy in *Pten*‐loss mouse models of PCa 10.

In this study, we hypothesized that treatment of prostate tumors with a combination of Pter and SAHA could be a more effective and less toxic way to target MTA1‐associated angiogenesis. We identified the direct link between MTA1 and HIF‐1*α* and the therapeutic potential of combinatorial approach to target MTA1/HDAC/HIF‐1*α*. Our preclinical findings demonstrate specific and potent anti‐MTA1/HIF‐1*α* activity of Pter/SAHA combinatorial treatment in prostate‐specific *Pten*‐null mice.

## Materials and Methods

### Reagents

Pterostilbene was synthesized following previously published procedures, and the purity of pterostilbene was determined to be >99% 27. Stock solutions of Pter were made using high‐purity dimethyl sulfoxide (DMSO) (Fisher Scientific, USA) and kept at 4°C, in the dark. SAHA was purchased from Selleckchem, USA. For injections, Pter (10 mg/kg bw) was freshly prepared daily in 10% DMSO. SAHA (50 mg/kg·bw) was suspended in solvent mixture containing DMSO, polyethylene glycol (PEG), and normal saline. Phytoestrogen‐free AIN 76A diet was obtained from Research Diets, USA.

### Cell culture

LNCaP cells (ATCC, USA) are hormone‐responsive nonaggressive PCa cells isolated from lymph nodes and they do not express PTEN tumor suppressor gene. PC3M cells (a gift from Dr. Bergan, Northwestern University, Chicago) are clonal variant of PC3 PCa cells initially isolated from bone metastatic lesion. These cells are very aggressive and they too do not express PTEN tumor suppressor. Both LNCaP and PC3M cell lines were maintained in RPMI‐1640 (ThermoFisher Scientific, USA) containing 10% FBS as described previously 7, 9, 10, 11, 12, 13, 14, 15 and kept in an incubator at 37°C with 5% CO_2_. Treatments with agents were carried out using phenol red‐free media containing 10% charcoal‐stripped serum as previously described 7, 8, 9, 10, 11, 12, 13, 14, 15. Cell lines were authenticated using short tandem repeat profiling at Research Technology Support Facility, Michigan State University, USA. Cells were tested for mycoplasma using the Universal Mycoplasma Detection Kit (ATCC, USA).

### MTA1 knockdown in PC3M prostate cancer cells

PC3M cells were transduced with the three GIPZ MTA1 lentiviral shRNAs, GIPZ GAPDH lentiviral shRNA as positive control, and GIPZ Non‐silencing (NS) lentiviral shRNA as negative control (GE Healthcare Dharmacon, USA). The GIPZ lentiviral shRNA system contains puromycin‐resistant gene for selection of transduced cells and TurboGFP for monitoring the selection under the fluorescence microscope. For preparation of the viral particles, we used the pCMV−ΔR8.91 packaging plasmid and the pMD.G envelope plasmid (Addgene, USA). Cells were transduced using RPMI medium (ThermoFisher Scientific, USA), which contained 4 *μ*g/mL polybrene (Sigma‐Aldrich, USA) with lentiviral particles at multiplicity of infection (MOI) = 8. On day 2 posttransduction, selection was initiated with 200 *μ*g/mL puromycin (Sigma‐Aldrich, USA) and GFP‐positive clones were selected and propagated.

### Cell viability assay

Cell viability was measured in LNCaP and PC3M cells after treatment with Pter and SAHA alone and in combination by MTT assay (Sigma‐Aldrich, USA) as described previously 8. Briefly, the cells were seeded in 96‐well plates and treated with compounds. Absorbance of the formazan was measured using BioTek Synergy‐4 plate reader (BioTek, USA) after 72 h of treatment. Percent of inhibition was calculated assuming no inhibition for DMSO‐treated control cells.

### Immunoblot analysis

Protein lysates from prostate tissues and cell lines were prepared in the RIPA buffer containing protease and phosphatase inhibitor cocktail (ThermoFisher Scientific, USA). Seventy microgram of protein was loaded in 10–15% SDS‐PAGE gel and transferred onto polyvinylidene difluoride (PVDF) membrane (Bio‐Rad, USA). Membranes were incubated in 5% nonfat dry milk/TBST blocking buffer for 1 h at room temperature, followed by an overnight incubation at 4°C in the presence of corresponding primary antibodies (Table S1). After washing with TBST, membranes were incubated in the presence of HRP‐conjugated secondary antibodies. Signal detection was performed using SuperSignal West Dura chemiluminescent substrate (ThermoFisher Scientific, USA). Quantitation was performed with ImageJ software (NIH, USA).

### ChIP‐Seq analysis

ChiP‐Seq experiments were performed as recently described by us 10. Briefly, mouse prostate tissues were used for MTA1 ChIP‐Seq at Active Motif Epigenetic Services (www.activemotif.com). The SICER algorithm was used for peak calling. Genome browser image of HIF‐1*α* gene (Fig. 3A) was generated using the UCSC Genome browser (http://genome.ucsc.edu).

### Quantitative RT‐PCR analysis

Total RNA was isolated from cell lines or homogenized prostate tissues using the RNeasy mini kit (Qiagen, USA). The quality of the RNA was determined on a Bio‐Rad Experion analyzer (Bio‐Rad, USA). RT‐PCR was performed on a CFX96 Real Time PCR Detection System (Bio‐Rad, USA) using the primer sequences given in Table S2, and fold changes in gene expression were determined as previously 10, 11, 12 using the 2^−ΔΔCt^ method 28. Experiments were performed in triplicates three separate times and shown as mean ± SEM.

### Mice breeding and genotyping

Female C57BL/6J mouse homozygous for the “floxed” allele of *Pten* gene was purchased from Jackson Laboratories, USA. B6Cg Pb‐Cre4 + (Cre+) male mouse was received from NCI mouse repository, USA. To be able to monitor spontaneous tumor development and progression in live animals, we utilized ROSA26‐pCAGGs‐LSL‐Luciferase mice, which were a kind gift from Dr. Azeddine Atfi (CI, UMMC, USA). After a series of carefully designed breeding strategies and genotyping, we collected 14 prostate‐specific luciferase (Luc) expressing *Pten* knockout male mice (*Pb‐Cre* ^*+*^ *; Pten* ^f/f^; *Rosa26* ^*Luc*/+^, hereafter referred as *Pten‐*null) for our experiments. We also collected 13 *Pten*‐null male mice without Luc expression for additional tissue collection.

Genotyping was conducted using tail genomic DNA by PCR‐based screening using PTEN‐, Cre‐, and Luc‐specific primers: PTEN geno olMR9554F:5′‐CAA GCA CTC TGC GAA CTG AG‐3′; PTEN geno olMR9555R:5′‐AAG TTT TTG AAG GCA AGA TGC‐3′ with a mutant band of 328 bp; Cre F: 5′‐TCG CGA TTA TCT TCT ATA TCT TCA G‐3′; Cre R: 5′‐GCT CGA CCA GTT TAG TTA CCC‐3′ with a band of 430 bp; and Luc F: 5′‐GCC ATT CTA TCC GCT GGA AG‐3′; Luc R: 5′‐GCT GCG AAA TGC CCA TAC TG‐3′ with a band of 332 bp (Fig. S1).

Animal housing, care, and treatments were in accordance with an approved protocol by Institutional Animal Care and Use Committee of UMMC. During the study, animals were permitted free access to drinking water and food, and were monitored daily for their general health.

### Treatment

Mice were randomized into four subgroups: vehicle (10% DMSO), treated with Pter (10 mg/kg·bw), treated with SAHA (50 mg/kg·bw), and with Pter + SAHA (10 mg/kg·bw plus 50 mg/kg bw). Intraperitoneal injections (i.p.) were performed daily, 5 days a week, for 10 weeks. The mice were sacrificed at 18 weeks of age, and their urogenital system (UGS) was isolated. Prostate tissues were used for RNA and protein isolation as well as for histological and immunohistochemical (IHC) analysis. Blood was also collected at the time of sacrifice, and serum samples were stored at −80°C until further analysis.

### Bioluminescence imaging of *Pten‐*null reporter mice

For this study, we generated prostate‐specific Luc‐reporter *Pten*‐null mice expressing Luc protein driven by the CAG promoter upon Cre‐mediated recombination (*Pb‐Cre* ^*+*^ *; Pten* ^f/f^; *Rosa26* ^*Luc*/+^). This allowed us to detect tumor‐specific luciferase expression in the prostate by performing noninvasive whole‐body imaging using the IVIS Spectrum System (Perkin Elmer, USA). Bioluminescent measurements for quantitative *in vivo* evaluation were taken every 4 weeks as previously described by us for xenografts 7, 9, 11, 12, 14. Briefly, mice were anesthetized by 2% isoflurane and injected i.p. with D‐luciferin (125 mg/kg, Perkin Elmer). Anesthetized mice were placed in the light‐tight box of the IVIS system for 10 min and whole‐body 1‐min images were acquired with medium binning. Image analysis and bioluminescent quantification was performed using Living Image software (Perkin Elmer, USA).

Cre‐negative (*Pb‐Cre* ^−^ *; Pten* ^f/f^; *Rosa26* ^*Luc*/+^) mice were used as normal prostate controls and Cre‐positive, *Pten* wild‐type (*Pb‐Cre* ^*+*^ *; Pten* ^*+/+*^; *Rosa26* ^*Luc*/+^) mice were used as luciferase background controls. Furthermore, the extent of light emission was dependent on whether one allele or two alleles of Luc were present. In this study, we used mice with one Luc allele.

### Histopathology and immunohistochemistry

Tissue paraffin embedding, sectioning, and H&E staining were performed by Reveal Biosciences Inc, USA. Four micrometer sections were prepared from formalin‐fixed paraffin embedded tissues and mounted on slides. Histological sections were prepared by hematoxylin and eosin (H&E) staining and were evaluated by pathologists (JRL, JML) who were blinded to the treatment. Immunohistochemistry was performed as described previously 7, 10, 11, 12, 14 using antibodies against CK8, SMA, Ki‐67, cleaved caspase‐3, CD31, MTA1, and HIF‐1*α* (Table S1), and the Vectastain ABC Elite Kit and ImmPACT DAB kit (Vector Laboratories, USA). Images were viewed and recorded using Nikon Eclipse 80i microscope (Nikon, USA). The ImageTool software was used to count positively stained cells in five randomly selected fields for each slide.

### ELISA

Conditioned media from cell lines or mice sera diluted in carbonate coating buffer (pH 9.6) was used to coat 96‐well plates, which were then incubated overnight at 4°C. Plates were then washed thrice with PBST (phosphate‐buffered saline with 0.05% Tween‐20) and blocked with 2% bovine serum albumin for 1 h at room temperature. Plates were then incubated with primary antibody (Table S1) diluted in blocking solution for 2 h at room temperature followed by HRP‐conjugated secondary antibody for 1 h at room temperature. Detection was done using the OPD (o‐phenylenediamine dihydrochloride) tablets (Sigma‐Aldrich, USA) and the end product was measured at 492 nm using BioTek Synergy‐4 plate reader (BioTek, USA). Experiments were performed in triplicates three separate times and shown as mean ± SEM.

### Tissue microarray analysis

The PCa tissue microarray (TMA) used in this study was constructed at the UMMC. Baseline characteristics of the patients in the TMA are shown in Table S3. The TMA was stained according to the IHC protocol described above and analyzed as described previously 29, 30.

### Patient sample analysis

Upon approval of the study from the Institutional Review Board (IRB, UMMC), fresh prostatectomy specimens were obtained immediately after surgery (CRP). The pathologists (IA, JCH) took core needle biopsies under sterile conditions, using Coaxial Achieve Automatic Biopsy System (14G × 11 cm) (CareFusion, USA) from tumor sites previously identified by biopsies and imaging studies. The tissues were immediately deep‐frozen for RNA isolation to perform qRT‐PCR using primers for MTA1 and HIF‐1*α* (Table S2). The qRT‐PCR data were then analyzed for correlation between MTA1 and HIF‐1*α* expression.

### Dataset analysis

Prostate cancer patient datasets available in the Oncomine database were queried for MTA1 and HIF‐1*α* expression. The Grasso et al. 31. dataset was then further analyzed by Microsoft Excel for correlation between MTA1 and HIF‐1*α* expression.

### Statistical analysis

The differences between the groups were analyzed by one‐way or two‐way ANOVA. The Fisher's exact test was used to evaluate the effect of treatments on mPIN incidence. Chi‐square test was used to evaluate the significance in TMA analysis. Statistical significance was set as *p *<* *0.05.

## Results

### Combination of Pter and SAHA effectively diminishes tumor growth in prostate‐specific *Pten*‐null mice

**Figure 1:**
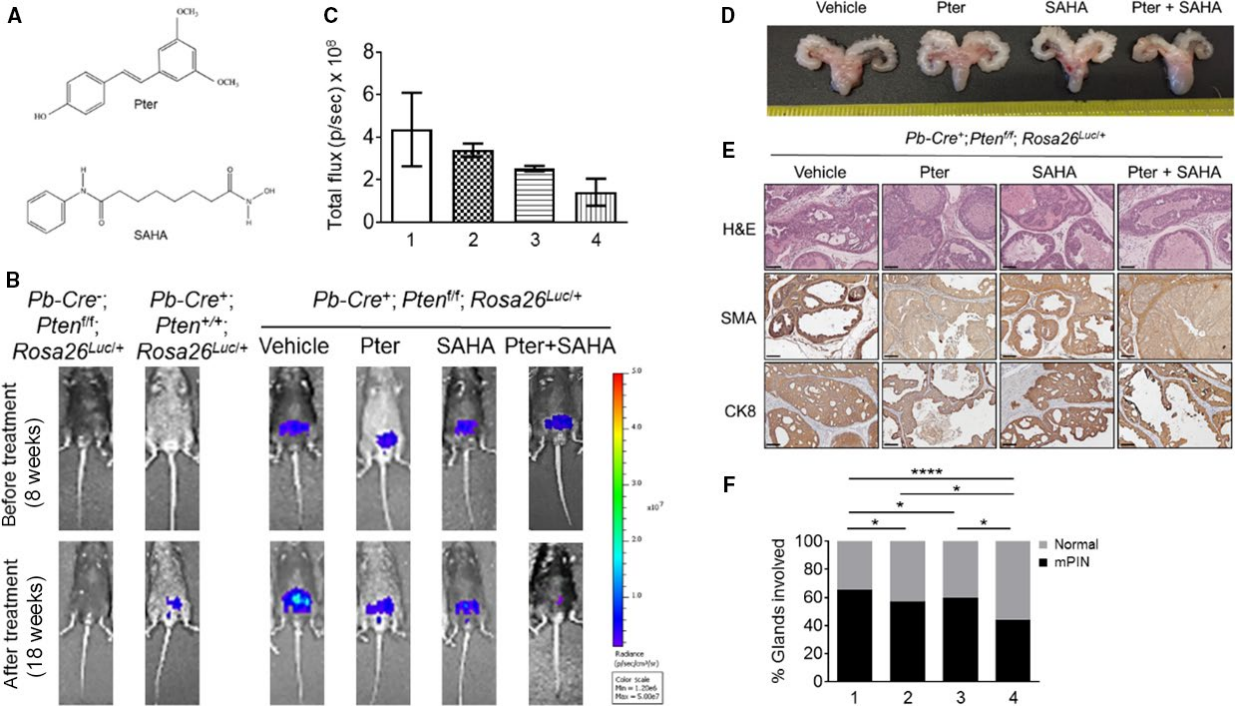
Combination of Pter and SAHA effectively diminishes tumor growth in *Pten*‐null mice. (A) Chemical structures of Pter and SAHA. (B) Representative images of luciferase reporter activity in the prostate of *Pten*‐null mice at 8 weeks of age (before treatment) and at 18 weeks of age (after treatment) are shown. *Pten*‐null mice were treated with vehicle (10% DMSO), Pter (10 mg/kg), SAHA (50 mg/kg), and Pter + SAHA (10 mg/kg and 50 mg/kg) by i.p. injections for 10 weeks. *Pb‐Cre* ^−^; *Pten* ^f/f^; *Rosa26* ^*Luc*/+^ mice were used as genetic background controls and *Pb‐Cre* ^*+*^; *Pten* ^*+/+*^; *Rosa26* ^*Luc*/+^ (*Pten* wild‐type) as luciferase background controls. (C) Quantitative analysis of tumor light emission in total flux (p/s) is plotted for each group of mice treated with (1) vehicle (*n* = 4), (2) Pter (*n* = 4), (3) SAHA (*n* = 3), and (4) Pter + SAHA (*n* = 3). (D) Gross anatomy images of urogenital system (UGS) of the representative mice, treated with vehicle, Pter, SAHA, and Pter + SAHA (dosage as above) for 10 weeks. (E) H&E‐stained sections and IHC analyses of SMA and CK8 in prostate tumors of *Pten*‐null mice at 18 weeks of age. Scale bar, 100 *μ*m. (F) Percentage of prostate glands involved in mouse PIN (mPIN) from 18 weeks old *Pten*‐null mice treated with (1) vehicle (*n* = 6), (2) Pter (*n* = 5), (3) SAHA (*n* = 5), and (4) Pter + SAHA (*n* = 7). *p <* *0.05; ****p <* *0.0001 (Fisher's exact test).

Generation of prostate‐specific luciferase reporter *Pten*‐null mice (*Pb‐Cre* ^+^; *Pten* ^f/f^; *Rosa26* ^*Luc*/+^) (Fig. S1) enabled monitoring tumor development and progression by direct luciferase detection using noninvasive whole‐body bioluminescent imaging. The antitumor activity of Pter and SAHA (Fig. 1A) alone as well as in combination was examined in cancer‐prone prostate‐specific *Pten*‐null mice during 10 weeks of treatment. Mice were treated daily with either Pter (10 mg/kg·bw) alone, SAHA (50 mg/kg·bw) alone, or combinations of Pter and SAHA (10 mg/kg·bw and 50 mg/kg·bw, respectively) by i.p. injections. Tumor growth was monitored by bioluminescent imaging every 4 weeks until 18 weeks of age when mice were killed and prostate tissues and sera collected for analysis. Luciferase measurements revealed tumor progression in vehicle‐treated control animals compared to the agent‐treated groups (Fig. 1B). While Pter alone and SAHA alone groups showed some tumor regression (a reduction of luciferase signal toward the end of the treatment), combined daily administration of Pter and SAHA considerably decelerated tumor growth in mice (Fig. 1B and C). The toxicity of treatments was assessed by observing behavior, monitoring body weight, and by necropsy at the conclusion of the study. Gross anatomy of UGS clearly indicated smaller prostates in agent‐treated mice, specifically in mice treated with Pter and SAHA combination, compared to vehicle‐treated control mice (Fig. 1D). All mice by 18 weeks of age developed high‐grade mouse prostatic intraepithelial neoplasia (mPIN) characterized by disorganized glandular structures with hypercellularity, which also retained CK8‐positive luminal cells and a basal layer of SMA‐positive cells (Fig. 1E). However, mice on treatments showed more favorable histopathology with restored normal ductal structures, as evident by H&E staining (Fig. 1E). Notably, mice on Pter and SAHA combination treatment exhibited drastic reduction (*p* < 0.0001) in the number of glands involved in mPIN compared to control mice and, more importantly, statistically significant reduction (*p *<* *0.05) compared to SAHA or Pter alone groups (Fig. 1F).

### Combination of Pter and SAHA reduces proliferation and angiogenesis, and promotes apoptosis *in vitro* and *in vivo*

**Figure 2:**
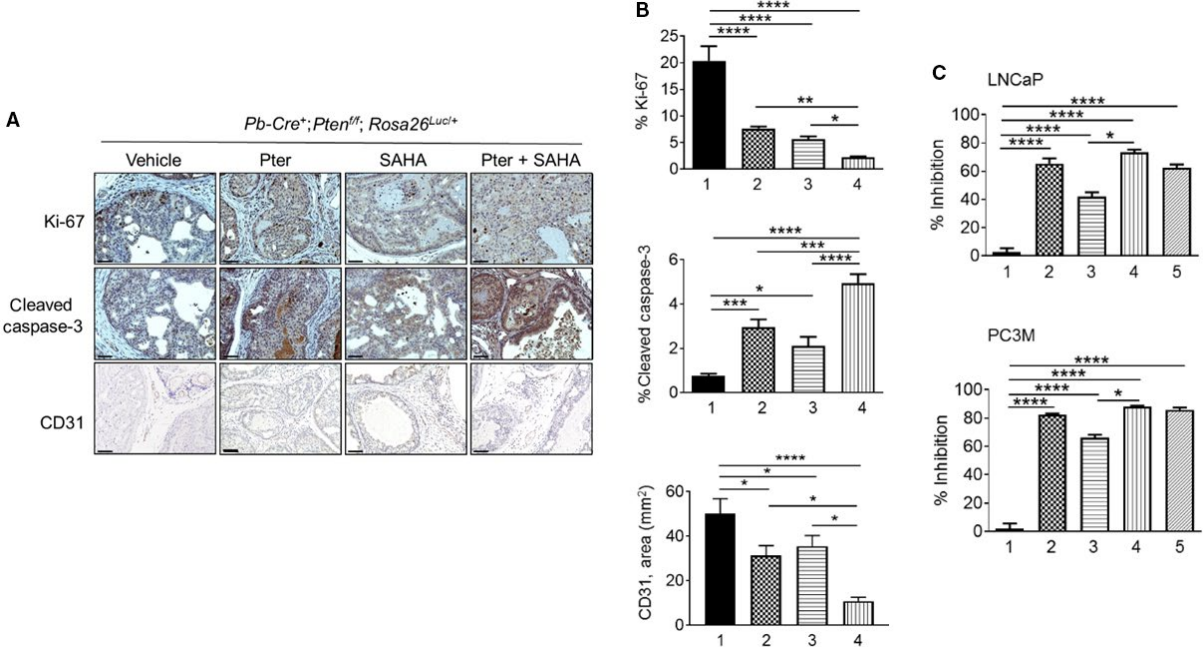
Combination of Pter and SAHA significantly inhibits cell proliferation and angiogenesis and induces apoptosis. (A) Representative Ki‐67 (*top, each panel*), cleaved Caspase‐3 (*middle, each panel*), and CD31 (*bottom, each panel*) staining of the prostate tissues from *Pten*‐null mice treated with vehicle (10% DMSO), Pter (10 mg/kg), SAHA (50 mg/kg), and Pter + SAHA (10 mg/kg and 50 mg/kg) by i.p. injections for 10 weeks. Scale bars, 50 *μ*m (Ki‐67, cleaved Caspase‐3); 100 *μ*m (CD31). (B) Quantitation of the proliferative (Ki‐67, *top*) and apoptotic (cleaved Caspase‐3, *middle*) indices and CD31‐positive areas (*bottom*) in *Pten*‐null mice. Mice were treated with (1) vehicle, (2) Pter, (3) SAHA, and (4) Pter + SAHA. Data are mean ± SEM (*n* = 3–6 mice/group), *p <* *0.05; **p <* *0.01; ***p <* *0.001; ****p <* *0.0001 (one‐way ANOVA). (C) Cell viability analysis of LNCaP (*top*) and PC3M (*bottom*) prostate cancer cells treated with (1) vehicle (10% DMSO), (2) Pter (50 *μ*mol/L), (3) SAHA (10 *μ*mol/L), (4) Pter + SAHA (50 and 5 *μ*mol/L), and (5) Pter + SAHA (50 and 10 *μ*mol/L). Data represent the mean ± SEM of three independent experiments. *p <* *0.05; ****p <* *0.0001 (one‐way ANOVA).

To assess the functional consequences of treatments, we analyzed tumor tissues for proliferation (Ki‐67) and apoptosis (cleaved Caspase‐3). The agent‐treated tumors had significantly lower levels of Ki‐67 and higher levels of cleaved Caspase‐3 compared to vehicle‐treated control mice (Fig. 2A). Particularly, there was a 10‐fold decrease in Ki‐67 staining and 10‐fold increase in cleaved Caspase‐3 staining in tumors treated with Pter/SAHA combination compared to control mice. Most importantly, statistically significant differences were seen when comparing combination treatment to SAHA and Pter alone (Fig. 2B, *top and middle*). In addition, staining with CD31 showed reduced angiogenesis in tumors treated with Pter/SAHA combination compared to controls and to single treatment groups (Fig. 2B, *bottom*). We also validated the antiproliferative effects of treatments alone and in combination *in vitro* using LNCaP and PC3M PCa cells. Both Pter and SAHA alone inhibited cell viability by approximately 60% and 40% in LNCaP and by 80% and 65% in PC3M cells at a concentration of 50 *μ*mol/L Pter and 10 *μ*mol/L SAHA, respectively (Fig. 2C, *top and bottom*). Notably, combination of Pter (50 *μ*mol/L) and SAHA at low 5 *μ*mol/L concentration (bar 4) showed significantly better effect on growth inhibition of LNCaP and PC3M cells compared to SAHA alone at high 10 *μ*mol/L dose (bar 3) (*p *<* *0.05). Interestingly, PC3M aggressive cells with higher metastatic potential were more sensitive to combined treatments than androgen‐sensitive LNCaP cells (80% vs. 60%). Taken together, our data indicate that Pter enhanced the sensitivity of PCa cells to SAHA. This suggests that chemosensitization by Pter to increase effectiveness of lower doses of SAHA may have potential benefit for therapeutic strategies in PCa.

### Combination of Pter and SAHA effectively inhibits MTA1/HIF‐1*α* axis *in vitro* and *in vivo*

**Figure 3:**
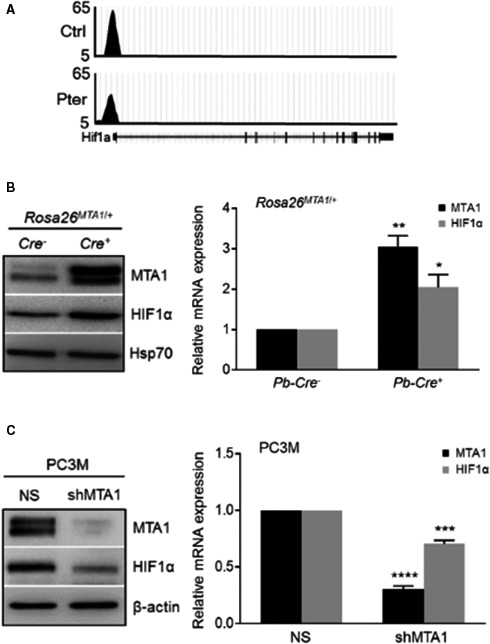
MTA1 directly regulates HIF‐1*α*. (A) Representative ChIP‐Seq track for HIF‐1*α* gene loci at 10 kb resolution [comparative analysis of MTA1 binding in the prostate tissues of mice on control and Pter supplemented diet 10]. (B) Immunoblot (*left*) and qRT‐PCR (*right*) analyses of MTA1 and HIF‐1*α* expression in prostate epithelium of wild‐type (Cre^‐^) and prostate‐specific MTA1‐transgenic mice (MTA1‐tg, Cre^+^) at 13 weeks of age. Hsp70 was used as a loading control. Data represent the mean ± SEM of three independent experiments. *p <* *0.05; **p <* *0.01 (one‐way ANOVA) compared to control Cre^−^. (C) Immunoblot (*left*) and qRT‐PCR (*right*) analyses of MTA1 and HIF‐1*α* expression in PC3M cells expressing MTA1 (NS) and silenced for MTA1 (shMTA1). *β*‐actin was used as a loading control. Data represent the mean ± SEM of three independent experiments. ***p <* *0.001; ****p <* *0.0001 (one‐way ANOVA) compared to NS control.

Our earlier studies using Pter treatment in PCa had shown potent inhibition of MTA1 *in vitro* and *in vivo* 7, 10. Since MTA1 often acts in complex with HDAC1 and 2, we hypothesized that combination with HDAC inhibitor (SAHA), which also downregulates MTA1 12, may selectively target MTA1/HDAC unit thereby regulating NuRD‐dependent cell survival and migration activity. As our previous studies revealed a close connection between MTA1 and angiogenesis 10, 14, we mined our MTA1 ChIP‐Seq data 10 for novel MTA1‐associated proangiogenic factors. We identified HIF‐1*α* as a downstream transcriptional target for MTA1 as revealed by decreased MTA1 occupancy of HIF‐1*α* promoter in Pter‐treated prostate tissues (Fig. 3A). Further, we found significantly high protein and mRNA levels of HIF‐1*α* concomitant with increased MTA1 levels in the prostate tissues from the prostate‐specific MTA1‐overexpressing transgenic mice (*Pb‐Cre* ^+^; *Rosa26* ^*MTA1*/+^) (Fig. 3B). Moreover, MTA1 depletion (shMTA1) in PC3M cells led to significant reduction of HIF‐1*α* protein and mRNA levels compared to cells transduced with non‐silencing (NS) control (Fig. 3C). Therefore, in addition to already known posttranslational regulation of HIF1‐*α* protein levels by MTA1 32, 33, we established a direct transcriptional regulation of HIF‐1*α* by MTA1 in PCa.

**Figure 4:**
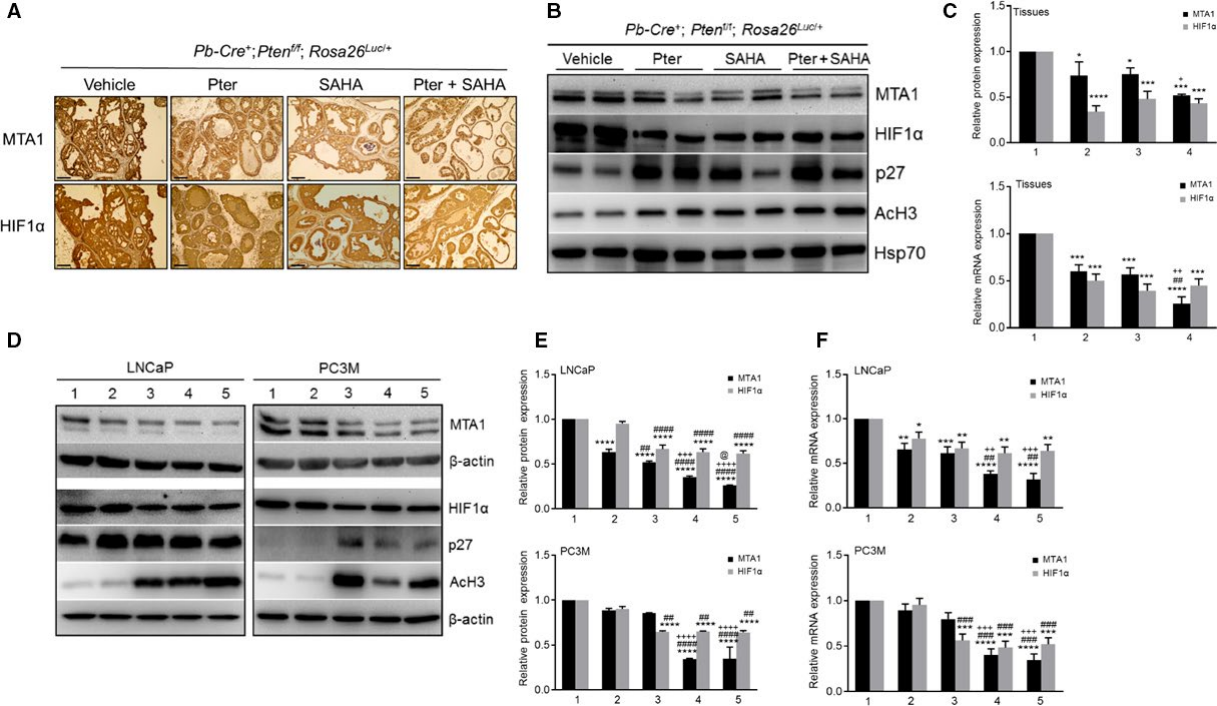
Inhibition of MTA1 and HIF‐1*α* by Pter and SAHA. (A) Representative MTA1 and HIF‐1*α* staining of the prostate tissues from *Pten*‐null mice treated with vehicle (10% DMSO), Pter (10 mg/kg), SAHA (50 mg/kg), and Pter + SAHA (10 mg/kg and 50 mg/kg) by i.p. injections for 10 weeks. Scale bars, 100 *μ*m. (B) Immunoblot analysis of MTA1, HIF‐1*α*, p27, and AcH3 in the prostate tissues from *Pten*‐null mice treated with vehicle, Pter, SAHA, and Pter + SAHA (dosage as above) for 10 weeks. Hsp70 was used as a loading control. (C) Quantitation of relative expression of MTA1 and HIF‐1*α* protein levels in prostate tissues from (B) (*top*); Quantitative RT‐PCR analysis (*bottom*) of MTA1 and HIF‐1*α* in the prostates from *Pten*‐null mice treated with (1) vehicle, (2) Pter, (3) SAHA, and (4) Pter + SAHA (dosage as above) for 10 weeks. Data represent the mean ± SEM of three independent experiments. *p <* *0.05; ***p <* *0.001; ****p <* *0.0001 compared to vehicle control; ^##^ *p* < 0.01 compared to Pter alone; ^+^ *p* < 0.05; ^++^ *p* < 0.01 compared to SAHA alone (2‐way ANOVA). (D) Immunoblot analysis of MTA1, HIF‐1*α*, p27, and AcH3 in LNCaP (*left*) and PC3M (*right*) prostate cancer cells treated with (1) vehicle (10% DMSO), (2) Pter (50 *μ*mol/L), (3) SAHA (10 *μ*mol/L), (4) Pter + SAHA (50 and 5 *μ*mol/L), and (5) Pter +SAHA (50 and 10 *μ*mol/L). *β*‐actin was used as a loading control. (E) Quantitation of relative expression of MTA1 and HIF‐1*α* protein levels from (D). (F) Quantitative RT‐PCR analysis of MTA1 and HIF‐1*α* mRNA expression in LNCaP (top) and PC3M (bottom) prostate cancer cells treated with (1) vehicle (10% DMSO), (2) Pter (50 *μ*mol/L), (3) SAHA (10 *μ*mol/L), (4) Pter + SAHA (50 and 5 *μ*mol/L), and (5) Pter +SAHA (50 and 10 *μ*mol/L). Data represent the mean ± SEM of three independent experiments. *p <* *0.05; **p <* *0.01; ***p <* *0.001; ****p <* *0.0001 compared to vehicle control; ^##^ *p* < 0.01; ^###^ *p* < 0.001; ^####^ *p* < 0.0001 compared to Pter alone: ^++^ *p* < 0.01; ^+++^ *p* < 0.001; ^++++^ *p* < 0.0001 compared to SAHA alone (two‐way ANOVA).

Next, we evaluated response to treatments by measuring the expression levels of MTA1 and HIF‐1*α* in tumor tissues (Fig. 4). The expression of both MTA1 and HIF‐1*α* at protein and mRNA levels in prostate tumor tissues from mice treated with agents was significantly reduced compared to vehicle controls (Fig. 4A–C). However, combined Pter/SAHA treatment showed significant decrease in MTA1 protein and mRNA but not in HIF‐1*α* levels, compared to SAHA alone. Further, since we have previously shown p27 as a transcriptional target of MTA1 10, we found upregulation of p27 levels concomitant with MTA1 decrease in tumors upon treatments. As an indication of HDAC inhibitory activity of our agents, we detected upregulated acetylated histone H3 (AcH3) levels in treated mice compared to vehicle controls (Fig. 4B).

Our *in vitro* experiments using LNCaP and PC3M cells treated with agents alone and in combination for 24 h showed downregulation of MTA1 and HIF‐1*α* compared to control cells further confirming our *in vivo* observations (Fig. 4D). Results showed that combination treatment with Pter at 50 *μ*mol/L and SAHA at low 5 *μ*mol/L (bar 4) demonstrate statistically significant reduction in MTA1 levels compared to SAHA alone at higher 10 *μ*mol/L concentration (bar 3) (Fig. 4E and F). Although there was no difference in HIF‐1*α* levels between the combination and SAHA treatment alone (bar 4 vs. bar 3), there was a significant reduction in HIF‐1*α* in cells treated with Pter/SAHA combination compared to Pter alone (bar 4 vs. bar 2) (Fig. 4E and F). Once again, as observed *in vivo*, p27 and Ac‐H3 were upregulated upon treatments in these cell lines.

Collectively, this data demonstrate a more potent MTA1/HIF‐1*α*‐targeted response to Pter/SAHA combination treatment than to single therapies both in murine prostate tumors and in PCa cell lines. This emphasizes that MTA1‐targeted chemosensitization by Pter to SAHA may have potential benefit for combination strategies versus monotherapies, in which downstream HIF‐1*α* is affected by SAHA but not by Pter.

### Pter/SAHA combination affects MTA1‐associated secretory proangiogenic factors

**Figure 5:**
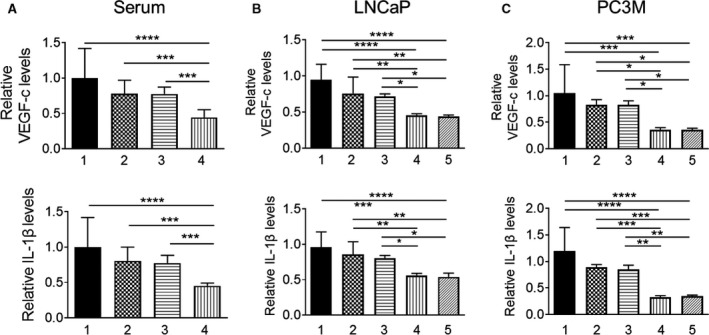
Pterostilbene and SAHA downregulates VEGF‐c and IL‐1*β* levels in murine serum and prostate cancer cells. (A) Circulating levels of VEGF‐c (*top*) and IL‐1*β* (*bottom*) in sera collected from *Pten*‐null mice treated with (1) vehicle (10% DMSO;* n* = 6), (2) Pter (10 mg/kg; *n* = 5), (3) SAHA (50 mg/kg; *n* = 5), and (4) Pter + SAHA (10 mg/kg and 50 mg/kg; *n* = 7) by i.p. injections for 10 weeks were quantitatively analyzed by ELISA. (B) Levels of VEGF‐c (*top*) and IL‐1*β* (*bottom*) were determined in conditioned culture media from LNCaP and (C) PC3M prostate cancer cells treated with (1) vehicle (10% DMSO), (2) Pter (50 *μ*mol/L), (3) SAHA (10 *μ*mol/L), (4) Pter + SAHA (50 and 5 *μ*mol/L), and (5) Pter + SAHA (50 and 10 *μ*mol/L). Plots represent relative levels acquired from three independent experiments. *p <* *0.05; **p <* *0.01; ***p <* *0.001; ****p <* *0.0001 (one‐way ANOVA).

To evaluate the effects of treatments on downstream proangiogenic effector molecules of MTA1/HIF1‐*α* axis, we determined IL‐1*β* and VEGF‐c levels in mouse sera and in conditioned media from PCa cells using ELISA. Our results showed that Pter/SAHA combination treatment was more effective in suppressing VEGF‐c and IL‐1*β* circulating levels compared to vehicle control and each agent alone (Fig. 5A). We found similar statistically significant reduction of secreted VEGF‐c and IL‐1*β* levels in conditioned media of LNCaP and PC3M cells treated with Pter/SAHA combination compared to vehicle‐treated control and each agent alone (Fig. 5B and C). However, we did not detect differences in VEGF‐c and IL‐1*β* levels between low (5 *μ*mol/L) and high (10 *μ*mol/L) doses of SAHA used in combination with Pter. Together, these findings point to the credence of utilizing MTA1‐targeted combinatorial approaches for improved management of PCa by providing informative proangiogenic predictive biomarkers. In addition, detection of IL‐1*β*, which is also well‐recognized as a proinflammatory molecule, will be an indicator of antiinflammatory effects of the combination regimen for PCa.

### Clinical relevance of MTA1/HIF‐1*α* axis in prostate cancer

**Figure 6:**
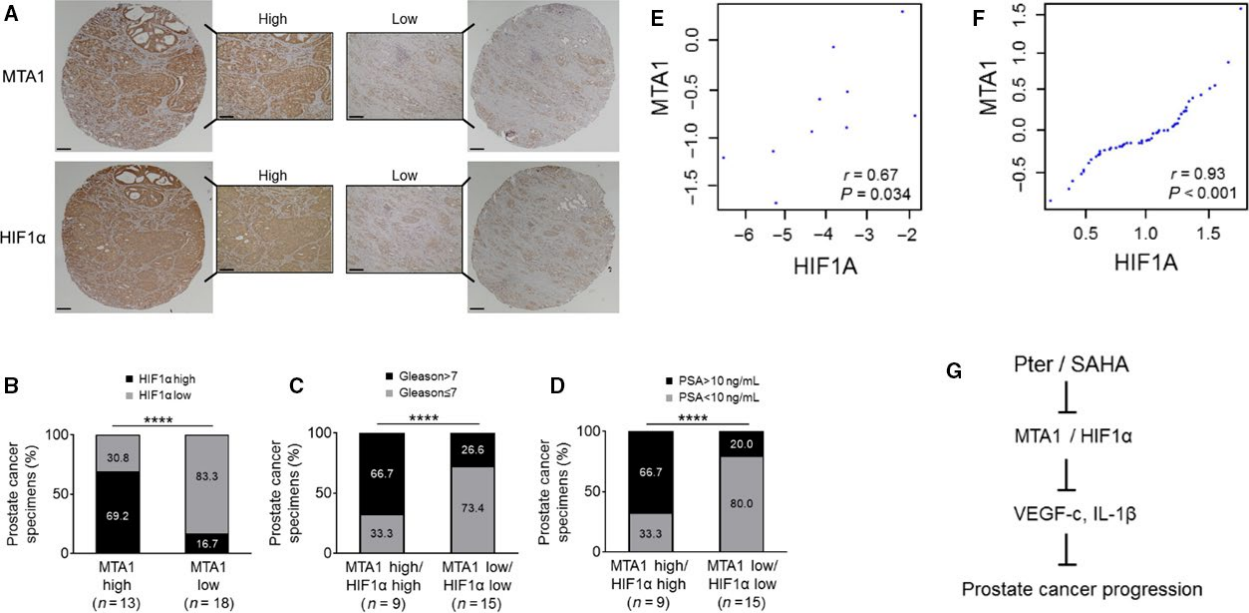
MTA1 and HIF‐1*α* expression positively correlate and indicate to poor outcome in prostate cancer. (A) Representative images of IHC staining of high (left) and low (right) MTA1 and HIF‐1*α* expression in prostate cancer TMAs. Scale bars, 500 *μ*m (whole core); 50 *μ*m (magnified image). (B) Quantitation of MTA1 and HIF‐1*α* staining showing that MTA1 and HIF‐1*α* demonstrated positive correlation in expression. (C) Correlation of MTA1 and HIF‐1*α* with Gleason score. (D) Correlation of MTA1 and HIF‐1*α* with preoperative PSA levels. ****p <* *0.0001 (Chi‐square test). (E) Positive correlation of MTA1 and HIF‐1*α* mRNA expression as determined in a cohort of 10 prostate adenocarcinoma biopsies. (F) Meta‐analysis showing the correlation between MTA1 and HIF‐1*α* levels from expression array^31^ using the Oncomine database. (G) Schematic diagram of proposed MTA1/HIF‐1*α*‐targeted effects of combinatorial Pter and SAHA treatment for preventing prostate cancer progression.

To further ascertain the clinical relevance of our findings, we examined MTA1 and HIF‐1*α* expression in our own custom‐made tissue microarrays with prostate tissues from 31 patients. We found that in MTA1‐high group, 69.2% showed high levels of HIF‐1*α*, whereas in MTA1‐low group, 83.3% showed low HIF‐1*α*, once again indicating positive relationship between MTA1 and HIF‐1*α* expression in prostate tumors (Fig. 6A and B). In addition, as expected, we observed positive correlation between high levels of MTA1/HIF‐1*α* that became stronger with higher Gleason scores and increased levels of prostate‐specific antigen (PSA), indicators of severity of the disease (Fig. 6C and D). Moreover, we found significant correlation between MTA1 and HIF‐1*α* mRNA expression (*r* = 0.67; *P* = 0.034) in our own 10 prostatectomy tumor samples obtained immediately after surgery (Fig. 6E). Further, we found a strong positive correlation between MTA1 and HIF‐1*α* expression in human PCa by analyzing data in the dataset 31 from the Oncomine database (Fig. 6F). Collectively, this data support a positive and strong correlation between MTA1 and HIF‐1*α* expression suggesting potential benefits of MTA1‐targeting antiangiogenic therapeutic strategies, particularly for advanced PCa.

## Discussion

We describe here a novel targeted combinatorial approach that consists of a dietary bioactive compound, Pter, and FDA‐approved anticancer drug, SAHA. The combinatorial approach may be a viable tool for management of cancer progression in certain subpopulation of PCa patients with high levels of MTA1.

The pathogenesis of PCa is characterized by dysregulation of different signaling pathways including overexpression of MTA1 5, 34, 35, 36, 37. We and others have shown that increased expression of MTA1 correlates with high Gleason score, aggressive disease, recurrence and bone metastasis 5, 36, 37. Our functional *in vitro* and *in vivo* studies demonstrated the role of MTA1 in promoting cancer cell invasiveness, angiogenesis, and metastatic potential 7, 10, 14.

On the molecular level, MTA1 complexes with HDAC1 and HDAC2 in NuRD corepressor complex and participates in the deacetylation of histones and certain nonhistone tumor suppressors 12, 15. As a “master‐co‐regulator” MTA1 plays a role in the recruitment of the complex to the promoter of specific genes for transcriptional silencing or transcriptional co‐activation 5, 38, 39, 40, 41, 42. Results from our MTA1 ChIP‐Seq analysis along with VEGF‐c and IL‐1*β* 10, identified HIF‐1*α* as a novel transcriptional target of MTA1 in PCa, emphasizing the importance of MTA1 in the promotion of cancer angiogenesis and metastasis. Others also have linked MTA1 with angiogenesis. Moon et al. showed stabilization and transcriptional activation of HIF‐1*α* due to deacetylation by MTA1/HDACs 32 and Yoo et al. indicated a close connection between HIF‐1*α*‐induced tumor angiogenesis and MTA1‐associated metastasis 33. In the current paper, we addressed the direct link between MTA1 and HIF‐1*α* and the therapeutic potential of combinatorial therapy to target MTA1/HDAC/HIF‐1*α*.

Combinatorial therapy, specifically with dietary bioactive compounds, is a very promising strategy for cancer management. Co‐application with approved anticancer agents may result in an additive or synergistic effect leading to enhanced anticancer efficacy. Importantly, this approach may decrease toxic side effects by lowering the effective dosage of anticancer drugs.

Pterostilbene, a potent natural analog of resveratrol found in blueberries, has been intensively tested by our group as MTA1‐targeted chemoprevention and therapeutic agent in PCa 2, 5, 6, 7, 10. As we had observed that Pter alone could prevent and block MTA1‐associated PCa progression in *Pten*‐loss mouse models 10, we rationalized whether inhibition of MTA1/HDAC/HIF‐1*α* by combination treatment of Pter with HDAC inhibitor SAHA may result in more powerful beneficial effect.

For this study, we generated prostate‐specific *Pten* knockout mice expressing luciferase reporter that allowed us to perform noninvasive *in vivo* whole‐body bioluminescent imaging. Our data show that tissue‐specific inducibility of the luciferase reporter is reliable in prostate‐specific *Pten*‐null model of PCa to monitor tumor progression and effects of treatments in real time.

We demonstrated that animals treated with combination of Pter and SAHA for 10 weeks had less tumor growth than mice treated with each agent alone, although due to limited number of mice in each group and interindividual variability of luciferase signal, we were unable to gather sufficient data to make for statistically significant differences. However, histological and IHC studies revealed more favorable histopathology, and showed significantly decreased tumor cell proliferation and increased apoptosis in mice treated with Pter and SAHA combination compared to mice treated with each agent alone. Consistent with our previous reports, while Pter treatment alone led to decreased angiogenesis in tissues (CD31 staining), combination treatment showed highly significant (*p *<* *0.0001) reduction in vessel formation and secretion of VEGF‐c and IL‐1*β* levels in serum compared to all other groups. Mechanistically, we found that combination of Pter and SAHA treatment had potent anticancer activity through targeting MTA1 and HIF‐1*α*. *In vitro* studies showed that SAHA at low dose (5 *μ*mol/L) in combination with Pter (50 *μ*mol/L) could achieve similar or better functional and molecular effects compared to SAHA alone at high dose (10 *μ*mol/L) suggesting chemosensitization of cancer cells by Pter. Of note, the SAHA dose (50 mg/kg) used in combination with Pter in our animal experiments gives a human equivalent dose 43 of about 250 mg/daily, which is lower than the high single dose of 400 mg/daily used in a recent phase II clinical trial in patients with metastatic castrate‐resistant PCa that was associated with significant toxicities 26. Therefore, our data reveal that lower nontoxic doses of SAHA could be effective when used in combination strategy.

We observed positive correlation between MTA1 and HIF‐1*α* in various PCa patient cohorts. Analysis of TMA revealed correlation between high levels of MTA1/HIF‐1*α* and unfavorable clinico‐pathological parameters such as high Gleason score and increased PSA levels.

In summary, our results establish MTA1/HIF‐1*α* axis as a suitable target for combinatorial therapeutic strategies with safe dietary stilbene Pter and approved anticancer drug SAHA (Fig. 6G). To the best of our knowledge, our study is the first to address MTA1‐associated angiogenesis targeting via combination strategy with dietary compound *in vivo*. In addition to improved bioavailability of Pter compared to resveratrol 44, 45, 46, 47, chemosensitization of cancer cells by Pter in combinatorial setting may become one of the most promising targeted strategies for better clinical outcome in PCa. This may help to overcome toxicity associated with higher doses of anticancer monotherapy 26. Our study also suggests that proangiogenic molecules determined in the blood may serve as predictive markers in future clinical trials designed to test combinatorial strategies with dietary compounds. These findings provide a novel perspective on how various combination strategies with dietary stilbenes may have more effective but less toxic therapeutic effects by targeting MTA1‐associated angiogenesis, which can be responsible for PCa progression and metastasis.

## Conflict of Interest

None declared.

## Supporting information


**Figure S1.** Mice genotyping. Tail‐DNA obtained from mice was subjected to PCR using specific primers for *Pten*,* Cre* and *Luc* (see Materials & Methods). 1% agarose gel image with PCR‐products for *Pten* (mutant 328 bp; wt 156 bp); *Cre* (392 bp), and *Luc* (332 bp) is shown.
**Table S1.** List of antibodies.
**Table S2.** List of primers.
**Table S3.** Baseline characteristics of patients included in the TMA.
