# Supplementary material for: Targeting MTA1/HIF‐1α signaling by pterostilbene in combination with histone deacetylase inhibitor attenuates prostate cancer progression
Source: Cancer Med. 2017 Oct 10;6(11):2673–85. doi: 10.1002/cam4.1209 (PMC5673954; doi:10.1002/cam4.1209)
Supplement: Supplementary file 1 — Figure S1. Mice genotyping. Tail‐DNA obtained from mice was subjected to PCR using specific primers for Pten, Cre and Luc (see Materials & Methods). 1% agarose gel image with PCR‐products for Pten (mutant 328 bp; wt 156 bp); Cre (392 bp), and Luc (332 bp) is shown. Table S1. List of antibodies. Table S2. List of primers. Table S3. Baseline characteristics of patients included in the TMA. [file CAM4-6-2673-s001.pdf]

Supplementary Materials Legends

Supplementary Fig. 1. Mice genotyping. Tail-DNA obtained from mice was subjected to PCR using specific primers for Pten, Cre and Luc (see Materials & Methods). 1% agarose gel image with PCR-products for Pten (mutant 328bp; wt 156bp); Cre (392 bp), and Luc (332bp) is shown.

Supplementary Table 1. List of antibodies

Supplementary Table 2. List of primers

Supplementary Table 3. Baseline characteristics of patients included in the TMA

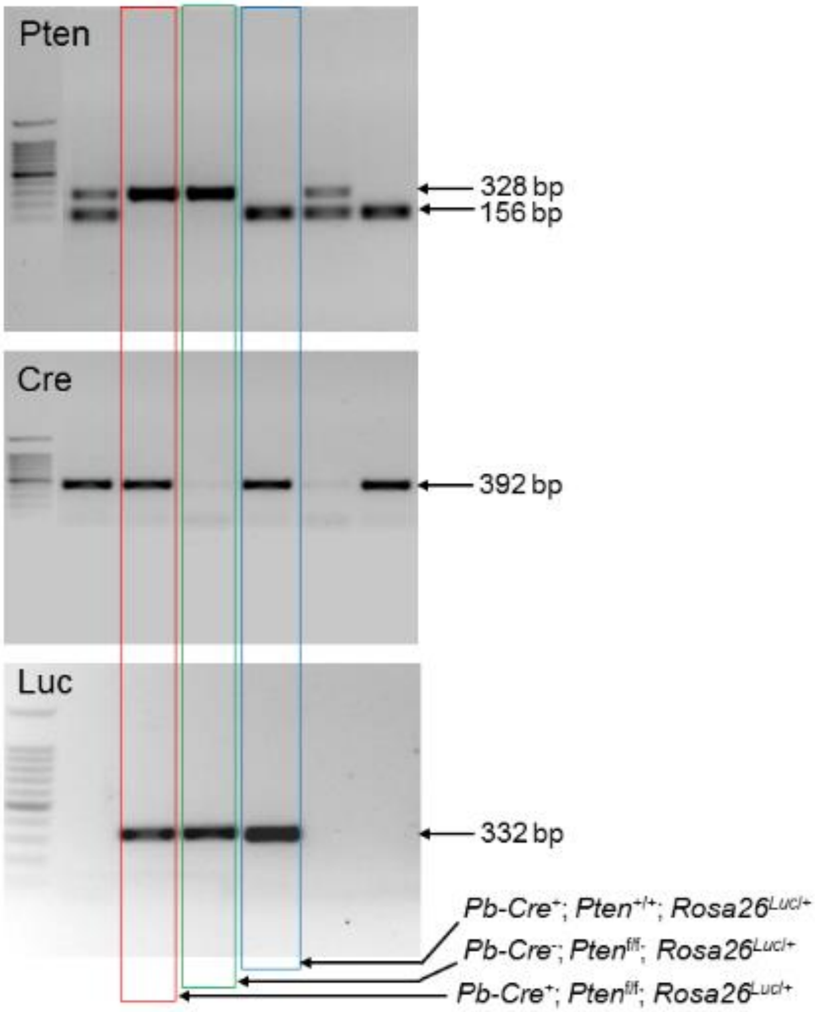
Pten
328 bp
156 bp
Cre
392 bp
Luc
332 bp
Pb-Cre+; Pten+/+; Rosa26Luc/+
Pb-Cre-; Ptenf/f; Rosa26Luc/+
Pb-Cre+; Ptenf/f; Rosa26Luc/+

Supplementary Table 1. List of antibodies

**Antibody**	**Catalog#**	**Company**	**Application**	**Dilution**
CK8	ab53280	Abcam	IHC	1:800
SMA	ab5694	Abcam	IHC	1:800
Ki-67	ab16667	Abcam	IHC	1:100
Cleaved Caspase 3	9664	Cell Signaling Technologies	IHC	1:100
CD31	NBP1-49805	Novus Biologicals	IHC	1:500
MTA1	5647	Cell Signaling Technologies	IHC<br>WB	1:50<br>1:1000
HIF1α	NB100-105	Novus Biologicals	IHC<br>WB	1:50<br>1:500
p27	sc-1641	Santa Cruz Biotechnologies	WB	1:200
Acetylated histone H3 (AcH3)	9649	Cell Signaling Technologies	WB	1:1000
Hsp70	sc-24	Santa Cruz Biotechnologies	WB	1:1000
β-actin	sc-69879	Santa Cruz Biotechnologies	WB	1:2500
IL-1β	sc-1250	Santa Cruz Biotechnologies	ELISA	1:50
VEGF-c	NB110- 61022	Novus Biologicals	ELISA	1:50

Supplementary Table 2. List of primers

Gene	Primers
m **MTA1**	**F:** 5′ CACTGGTGCTGAAGCAGGTA 3′<br>**R:** 5′ ACTGCTGAGCACACTGGATG 3′
h **MTA1**	**F:** 5′AGCTACGAGCAGCACAACGGGGT 3′<br>**R:** 5′CACGCTTGGTTTCCGAGGAT 3′
m **HIF1α**	**F:** 5′ GAAATGGCCCAGTGAGAAAA 3′<br>**R:** 5′ CTTCCACGTTGCTGACTTGA 3′
h **HIF1α**	F: 5′ TGCTCATCAGTTGCCACTTC 3′<br>R: 5′ TCCTCACACGCAAATAGCTG 3′
m **β-actin**	**F:** 5′GATCTGGCACCACACCTTCT 3′<br>**R:** 5′GGGGTGTTGAAGGTCTCAAA 3′
h **β-actin**	**F:** 5′CGTGGGCCGCCCTAGGCACCA 3′<br>**R:** 5′TTGGCTTAGGGTTCAGGGGGG 3′

m, mouse; h, human; F, forward primer; R, reverse primer

Supplementary Table 3. Baseline characteristics of the patients included in the TMA

**Clinical and pathologic characteristics of the patients (N=38)**	**N (%), range**	**African Americans N (%), range**	**Caucasians N (%), range**
Age, years	60.5 (45-81)	60.5 (50-69)	61 (45-81)
Race/Ethnicity			
Caucasians (%)	19 (50)		
African Americans (%)	19 (50)		
PSA at the time of surgery, ng dl^{-1}	7.1 (2.6-140)	8.7 (2.6-33)	7 (3.8-140)
Pathologic Gleason sum			
⩽6	17 (44.7)	11 (57.9)	06 (31.6)
7	10 (26.3)	4 (21.0)	06 (31.6)
>/= 8	11 (28.9)	4 (21.0)	07 (36.8)
Extracapsular extension (%)	09 (23.7)	05 (26.31)	04 (21.05)
Positive surgical margins (%)	05 (13.2)	02 (10.51)	03 (15.78)
